# Commentary: Ensuring health statistics in conflict are evidence-based

**DOI:** 10.1186/1752-1505-4-10

**Published:** 2010-05-05

**Authors:** Leslie F Roberts

**Affiliations:** 1 Program on Forced Migration and Health, Columbia University

## Abstract

The author argues that measuring mortality in conflict settings is fraught with limitations which mostly result in under-estimation of mortality. Some recent publications on this subject have been based upon convenient surveillance processes, or even press reports. The author calls for vigilance against such studies and argues that war related surveillance-based mortality estimates should include measures of sensitivity and representativeness.

## 

This January (2010), the second Human Security Report was released with much fanfare and an opening line stating, "...this report reveals that nationwide mortality rates actually fall during most wars." This conclusion which flies in the face of the entire humanitarian endeavor (designed to minimize excess mortality), can easily be dismissed as the artifact of a poorly done report. The report; defines a war as ongoing when only 25 killings per year are occurring, uses national surveys with multiyear recall periods to examine short minor conflicts rather than examining the conflict-affected populations, weighs minor conflicts and major wars as similar events, and selectively cites sources to make their points often ignoring the overall conclusions of those same sources. The report received limited credence in the press and even less in the academic community and hopefully will be quickly forgotten. What is important for those of us involved in the documentation of human suffering, is that this report is the latest and worst in a growing trend of non-public health professionals drawing health conclusions from convenient samples.

Since Karl Western made the first modern estimate of deaths during the Biafran conflict, the public health community has struggled to collect data that was evermore sensitive and representative [1]. From the earliest guidelines for displaced populations, public health officials have struggled against the under-reporting of deaths[2]. This chronic under-reporting, while particularly problematic with surveillance, also occurs in household surveys [3,4] Aside from the Human Security Report, whose conclusions are largely based on news media reports, a variety of other publications have been produced based on press reports, or worse, passive surveillance by governments involved in a war [5,6] This Journal has shown that news reports are in part a cultural construct. For example, the ratio of civilian to Coalition military deaths in Iraq reversed when comparing 11 US newspapers with three from the middle east[7]. The dangers of drawing conclusions from passive surveillance processes are profound: they allow one to conclude mortality goes down in times of war making war more acceptable, and they allow armies, like those invading Iraq, to manipulate the press to portray resistance fighters as the primary killers when population-wide data conclude the opposite [8,9].

Our public health struggle to constantly improve the sensitivity and representativeness of war-time morbidity and mortality information has slipped backwards in recent years. This has not happened because of poor work by our peers, it has happened by having professionals from other fields bring their new and insightful talents to bare in the areas of human rights and mortality without learning even the basics about health data collection. We should not tolerate publications of surveillance data where the sensitivity of that data cannot be shown. We should not allow ratios of numbers to define wrong doing in a field where rates are the basis for judgment. As governments become better at imbedding reporters, at controlling the internet, and banning foreign reporting, we need to become more outspoken about the weaknesses, abuse, and misapplication of health data. A 2007 Associated Press poll found that when asked how many of their soldiers had died in Iraq, Americans had a median estimate of 97% of the truth, but when asked about Iraqis, the median estimate was less than 2% of reality [10]. We who work with surveillance data and surveys, need to consider how does this level of ignorance get generated and what is our role in a democracy in correcting the record? If ever there was a message that needed to be corrected, "mortality rates actually fall during most wars" seems like a prime candidate.
